# Association between trusted sources of information and COVID-19 protective behaviors: results from a longitudinal survey of adults living in New York City during the pandemic

**DOI:** 10.3389/fpubh.2026.1838172

**Published:** 2026-09-09

**Authors:** Emily Gill, Lorna E. Thorpe, Donna Shelley, Natasha J. Williams

**Affiliations:** 1 Department of Population Health, NYU Grossman School of Medicine, New York, NY, United States; 2 Department of Public Health Policy and Management, NYU School of Global Public Health, New York, NY, United States; 3 Center for Healthful Behavior Change, NYU Grossman School of Medicine, New York, NY, United States

**Keywords:** COVID-19, health behavior, health promotion, information sources, trust

## Abstract

**Introduction:**

For the public to adopt health promoting behaviors widely, there has to be sufficient trust in the institutions that disseminate information on protective behaviors. However, when members of the public lose trust in formal information sources, many seek information from informal information sources. Identifying trusted informal messengers is key to restore trust and effectively promote public health.

**Methods:**

We conducted repeat surveys with young adults and older adults living in New York City public housing to assess who they consider a trusted source of COVID-19 information, as well as their protective behavior practices across a period of five weeks. We then examined the association between trusted sources and participant behavior.

**Results:**

Participants in both age groups showed the highest trust in doctors or healthcare providers. Associations with protective behavior existed across multiple information sources, but strongest associations for both age groups were seen in relation to trusting one’s faith leader for information.

**Discussion:**

Although other sources of information were more frequently cited as trustworthy by participants, trust in one’s faith leader was the strongest predictor of protective behavior practices.

## 1 Introduction

Amid pandemics and other public health crises, public health institutions play a central role in disseminating information, resources and recommendations. However, dissemination alone is insufficient. Broad adoption of recommended health promoting behaviors relies fundamentally on public trust in the institutions that disseminate them (1–6). Since the COVID-19 pandemic, numerous studies show that public trust in institutions has declined precipitously (6–8). Factors such as information overload, inconsistent messaging, and the perception that public health institutions are influenced by political agendas have contributed to this erosion of trust. As a result, many people rely on informal networks for information and decision-making (4, 9–11). Considering these shifts, it is important to gain a better understanding of groups or individuals that are perceived as trustworthy messengers, and how that trust shapes responses to such recommendations. Moreover, public health institutions have an opportunity to collaborate with trusted informal networks to rebuild public trust and promote effective strategies for optimizing health promotion and behavior.

This study analyzed data collected as part of a nationwide National Institutes of Health’s Rapid Acceleration of Diagnostics—Underserved Populations (RADx-UP) initiative. This initiative assessed inequities in COVID-19 testing and infection and evaluated strategies to increase testing uptake among New York City (NYC) residents living in public housing.

This paper presents findings from survey data collected between June 2022 and September 2023 that assessed who participants deemed trustworthy in providing information on COVID-19, and associations with protective behaviors measured across a five-week period. By analyzing these relationships, this research contributes to the growing body of evidence on trust, information dissemination, and health behaviors relating to pandemics.

## 2 Materials and methods

A total of 600 participants were enrolled in a two-arm, clustered randomized trial designed to evaluate different methods for improving reach of COVID-19 testing. Young adults (ages 16–29 years), and socially isolated older adults (ages 60 years and older), were targeted due to their distinct risk profiles—teenagers and young adults were major drivers of SARS CoV-2 transmission, whereas isolated older adults may not be easily reached by interventions. Moreover, these groups may experience public health messaging, social influence, and barriers to protective action in distinct ways: young adults often rely on peer and social media networks and may perceive lower personal risk, whereas socially isolated older adults may face reduced exposure to informal support and greater vulnerability to severe outcomes Further detail on the selection of these groups can be found elsewhere (12).

Individuals were referred to the study through community partners, who recruited participants using flyers, social media, and in-person outreach. Once enrolled, participants were provided with rapid antigen COVID-19 at-home test kits and asked to complete a baseline survey and five brief, weekly follow-up surveys. Surveys were administered in English, Spanish, and simplified Chinese. Surveys could be self-administered electronically or administered telephonically by a research team member. Participants were compensated for their time. The study protocol was approved by the Institutional Review Board of NYU Grossman School of Medicine (i20-01636).

For this analysis, we used the following question from the baseline survey to assess trust: “how much do you trust each of these sources to provide correct information about COVID-19?” Source options included: 1) doctor or health care provider, 2) faith leader, 3) close friends and members of their family, 4) people they go to work or class with, 5) news on the radio, TV, online, or in newspapers, 6) contacts on social media, 7) the U. S. government, and 8) the U. S. Coronavirus Task Force (USCTF). Responses included “not at all,” “a little,” “somewhat,” and “a great deal.” For our analysis, these responses were collapsed into “does not trust” and “does trust.” Performance of protective behaviors was reported using data from the weekly follow-up surveys. These included mask-wearing and participants’ use of the at-home test kits provided to them by the study. Results from the follow-up surveys were pooled across the 5 weeks, and performance of a given behavior was collapsed into a yes/no variable. Prevalence ratios were calculated to measure the strength of association between trust and performance of protective behaviors.

## 3 Results

In Table 1, we present the study participant demographics. Most older adults identified as Asian. In contrast, young adults largely identified either as Hispanic/Latino or non-Hispanic Black. Most study participants were English speakers.

**Table 1:** Demographics of NYC public housing resident survey participants, stratified by age group (2022–2023).

Sex	Overall%	Socially-isolated older adults 60 + years%	Young adults 16–29 years old%
	(*N* = 598)	(*N* = 449)	(*N* = 149)
Female	61.0%	64.2%	51.4%
Race/ethnicity			
Hispanic or Latino	28.7%	26.2%	36.2%
Non-Hispanic Black	24.0%	19.7%	36.9%
Asian	42.5%	50.8%	17.5%
Other*	4.9%	3.4%	9.4%
Language spoken			
English	41.35%	32.2%	69.1%
Spanish	23.71%	26.3%	15.8%
Chinese dialects	33.69%	41.5%	10.1%
Other	1.25%	0.0%	5.0%

*Other includes those who identify as American Indian or Alaska Native, Native Hawaiian or Pacific Islander, non-Hispanic White, and “Some other race”.

Table 2 summarizes the association between trust and use of protective behaviors. Among both older and young adults, a doctor or healthcare provider was the most trusted source of COVID-19 information. Older adults tended to trust the government, including the US Coronavirus Task Force, whereas trust in government as sources of information was lower among younger adults. Older and younger adults responded similarly across the other categories, though generally speaking older adults showed slightly higher trust in institutional sources of information (governmental, faith leader and traditional news media), and younger adults showed slightly higher trust in informal sources of information (family and friends, colleagues and social media).

**Table 2:** Prevalence ratios for performance of protective behaviors and trusted information sources among older and young adults, NYC (2022–2023).

Sources of Information		Doctor or health care provider		The U. S. coronavirus taskforce		The U. S. government		Close friends and family		Faith leader		News on the radio, tv, online, or in newspapers		People you go to work or class with		Your contacts on social media	
		SIOA *n* = 441	YA *n* = 137	SIOA *n* = 373	YA *n* = 124	SIOA *n* = 408	YA *n* = 124	SIOA *n* = 408	YA *n* = 129	SIOA *n* = 343	YA *n* = 113	SIOA *n* = 402	YA *n* = 128	SIOA *n* = 373	YA *n*= 128	SIOA *n* = 327	YA *n* = 128
Percent of participants that trust this source		95.0%	94.9%	83.9%	65.3%	79.2%	59.7%	76%	78.3%	69.4%	63.7%	60.2%	57.8%	59.5%	64.1%	35.5%	42.2%
Association between participant trust and performance of COVID-19 protective behaviors in past week (PR)	Wore a mask while at workplace, school, or volunteer site	0.93	0.74	1.03	**0.75**	0.99	0.85	1.06	0.88	**1.18**	0.97	0.99	0.86	1.06	0.89	0.99	1.12
	Wore a mask while at large social event such as concert, sports game, or dance	0.85	1.07	1.00	0.91	**1.19**	1.13	1.03	1.01	1.11	**1.91**	1.11	1.27	1.08	1.08	1.05	1.19
	Wore a mask while spending time socially with more than 5 people	0.91	0.92	**1.17**	0.85	**1.23**	1.18	1.07	1.16	**1.23**	**1.67**	1.06	0.93	1.06	0.97	1.05	1.07
	Wore a mask while in restaurant, coffee shop, or club	0.92	1.07	**1.29**	0.89	**1.34**	1.32	1.02	0.97	**1.15**	1.00	0.95	1.01	0.97	0.75	1.07	0.86
	Wore a mask while on the subway or bus	1.15	0.96	**1.13**	0.92	**1.11**	1.14	**1.09**	1.10	**1.07**	1.05	**1.06**	0.90	1.05	**0.80**	1.00	0.90
	Wore a mask while at store, supermarket, mall, or retail shopping	1.02	0.80	**1.15**	0.98	**1.12**	1.19	1.02	1.00	**1.05**	1.06	1.02	0.85	1.01	**0.81**	0.97	1.00
	Used an at-home COVID test	0.82	0.94	0.99	0.86	0.98	1.08	0.97	**1.34**	**1.25**	**1.37**	1.03	1.06	**1.22**	1.20	**1.11**	**1.25**

Bolded text indicates the finding was statistically significant (*p* < 0.05). In this table, green indicates a positive association, yellow indicates negative. Only significant results are bolded and highlighted.

YA, young adults (ages 16–29); SIOA, socially isolated older adults (ages 60+).

We found no association between trusting doctors or healthcare providers and protective behaviors in either age group. For older adults, reporting trust in the USCTF and the U.S. Government was positively associated with mask-wearing in various settings. Notably, younger adults who trusted the USCTF were less likely to wear a mask at large social events (PR 0.75). There was a marginal positive association between trusting close friends and family and mask-wearing on public transit for older adults (PR 1.09), and usage of at-home COVID test among younger adults (PR 1.34). Trust in faith leaders was significantly associated with multiple protective behaviors across both age groups. In addition, among young adults, trust in faith leaders was the only source of information associated with an increased likelihood of wearing a mask (1.91 times more likely to have worn a mask at a large social gathering, and 1.67 times more likely to have worn a mask while socializing with 5 or more people), and had the strongest correlation for both age cohorts for using an at-home COVID test (PR 1.25 for older adults, 1.37 for younger adults). Among older adults, trust in news media showed only a minor positive association with wearing masks on the subway or bus. Among participants who trusted coworkers or peers in school, older adults had an increased likelihood of using an at-home COVID test, whereas young adults showed a decreased likelihood of wearing a mask on public transit and while shopping (PR 0.80 and 0.81, respectively). Finally, trust in social media contacts showed no associations with mask-wearing, but was positively associated with at-home test usage in both age cohorts (PR 1.11 and 1.25, respectively).

## 4 Discussion

This study highlights relationships between trust in information and adoption of protective behaviors such as mask-wearing and testing. Our analysis showed that older and younger adults shared similar levels of trust across the different sources, except for lower trust of the U.S. Government and the USCTF among younger adults. Still, more than half in both age groups found these governmental bodies trustworthy, which is consistent with other studies (13, 14). Older adults exhibiting higher trust of formal institutional sources is in line with previous findings (15), as is our finding that young adults were slightly more trusting of social media (16).

Similar to other studies, our results showed that trust in governmental or public health institutions was correlated with mask-wearing, though this was only true of our older adult cohort (17, 18). However, some of our findings were contrary to what has been published elsewhere: one study found that institutional trust was positively associated with testing behavior, and another found that trust in the media was associated with increased masking, which we largely did not see in our own results, regardless of age group (17, 19). Also somewhat notable was our finding that, with the exception of trust in one’s faith leader, for younger adults trust had almost no association with mask-wearing, and in some cases there was a negative relationship.

Though mask wearing in general is associated with feelings of perceived risk, which may be lower among young adults, this does not explain the absence of a relationship between trust and mask-wearing among this group, which may even be counterintuitive given the fact masking is often seen as a pro-social activity and motivated by concern for others (17, 20). Perhaps, as one author notes, it could be because “social trust is agnostic to health promotion,” and perceived trustworthiness may lead people to drop their guard and forgo preventive measures such as masking (20). Further research should be done to explore this phenomenon.

Finally, our findings revealed that trust in one’s faith leader had the strongest correlations with protective behavior. Young adults who trusted their faith leaders were almost twice as likely to engage in mask-wearing in social settings and illustrated the highest likelihood of using an at-home COVID test. Similarly, older adults who trusted their faith leader showed a higher likelihood of mask-wearing across various settings and showed the highest likelihood of at-home COVID test.

The relationship between religion and COVID protective behavior is mixed, and in many cases increased religiosity is associated with decreased adherence to public health guidelines (21–24). One study found that having high trust in one’s faith leader for COVID related information was associated with lower rates of vaccination (25). However, faith leaders can also be leveraged to promote public health practices. Viskupic et al. (26) demonstrated that encouragement from a faith leader resulted in a substantive increase in intent to vaccinate, and that the effect was strongest among people younger than 65 years old.

Historically, faith-based organizations (FBOs) have shown to be valuable resources for public health interventions, including those related to HIV/AIDS, mental health, cancer screening and COVID-19 (27–34). Strengths of these institutions are their active and engaged memberships, robust communication infrastructure, and long-standing relationships with community members that engender trust and influence (28, 30). These institutions may also align with many of the social and moral tenets of public health, including a strong emphasis on community safety and well-being, making them ideal partners for public health crises (35, 36). Despite these strengths and a demonstrable willingness on the part of FBOs to engage in health promotion (37), collaborations between public health institutions and FBOs are often underutilized (29).

Findings from this study support the need to tailor communication and outreach strategies to diverse age groups, particularly in the context of rebuilding trust in public health and promoting protective health behaviors. A “one-size-fits-all” approach would be inadequate for complex health interventions, and results may be suboptimal unless trusted formal and informal networks are engaged.

This study has several limitations. For one, individuals were recruited by community-based organizations to take part in a study about COVID. As such, it is possible the individuals enrolled were 1) more trusting of institutions than the general population and 2) may also be more COVID conscious and thus likely to engage in protective behaviors. Additionally, the results may not be generalized outside of NYC, which was significantly impacted during the early waves of the pandemic. Also significant is the fact that we did not directly examine the social and contextual factors relevant to our study population. We acknowledge that public housing residents have long been affected by concentrated poverty, disinvestment, barriers to healthcare access, and differential exposure to health risks. During the COVID-19 pandemic, these health inequities may have been further exacerbated by crowded living conditions, limited access to linguistically and culturally responsive health information and uneven access to testing and care. Additionally, as they were not the focus of our research question, we did not conduct further analyses on variables apart from age, such as gender or race/ethnicity. These are important considerations in interpreting our findings and should be explored in future analyses. Nonetheless, this study contributes novel insights by revealing the relationship of trust and protective behaviors, whereas most existing literature focuses exclusively on vaccination. Moreover, our findings highlight an association between faith and performance of protective behaviors across a diverse population and irrespective of any (known) intervention, which provides a foundation for further research.

## Data Availability

The original contributions presented in the study are included in the article/Supplementary material, further inquiries can be directed to the corresponding author.
