# Supplementary material for: Association between trusted sources of information and COVID-19 protective behaviors: results from a longitudinal survey of adults living in New York City during the pandemic
Source: Front Public Health. 2026 Sep 9;14:1838172. doi: 10.3389/fpubh.2026.1838172 (PMC13597789; doi:10.3389/fpubh.2026.1838172)
Supplement: Supplementary file 1 [file Supplementary_file_1.docx]

Version date: 1/25/2023

Intake Survey

*Screening & Enrollment - NYCHA RCR Phase II*

*Page 1*

**Congratulations! You are eligible for a lottery where you can win $150 for your participation in the study. You will complete one survey when you sign-up and get a testing kit for COVID-19. You will use the test at-home. Next, we will send you 5 short surveys. We will send you one survey every week for 5 weeks.**

ID

Randomization	Arm 1 - Control

Arm 2 - Intervention

Please specify other:

Date of Data Collection

(MM/DD/YYYY)

**[CHW Instructions]**

As a part of the project, we ask all residents to complete a survey. In the survey, we will ask basic information about you, your health, and COVID-19. I will read each question to you. Remember, your answers are confidential. The survey will take approximately 25 minutes to complete. Are you ready to begin?

**Consent / Consentimiento**

Is consent required for this study?	Yes, consent is required for this study

No, Consent is not required/is waived for this study

Date of Consent

(MM/DD/YYYY)

**About You / Sobre usted**

Date of Identity Collection

First Name

Last Name

Street Address

Apartment Number

Street Address 2

City

Zip Code

County

State or Territory	Alabama

Alaska Arizona Arkansas California Colorado Connecticut Delaware

District of Columbia(DC) Florida

Georgia Hawaii Idaho Illinois Indiana Iowa Kansas Kentucky Louisiana Maine Maryland

Massachusetts Michigan Minnesota Mississippi Missouri Montana Nebraska Nevada

New Hampshire New Jersey New Mexico New York

North Carolina North Dakota Ohio Oklahoma Oregon Pennsylvania Rhode Island South Carolina South Dakota Tennessee Texas

Utah Vermont Virginia Washington West Virginia Wisconsin Wyoming

American Samoa GUAM

Northern Mariana Islands Puerto Rico

US Virgin Islands

Mobile Phone

Home Phone

Other Phone

Personal Email

Other Email

Preferred Method of Contact	Mobile phone Home phone Other phone Personal email Other email

Date of Birth

(MM/DD/YYYY)

Date of Sociodemographic Data Collection

**RCR Awareness / Concienciación sobre la respuesta de los residentes a la COVID (Resident**

**Covid Response, RCR)**

1. Where did you hear about this project?

From another resident From a tenant leader From NYCHA staff

From a local community organization (e.g. Henry Street Settlement, HCCI, CAMBA)

From a flyer

From a NYCHA communication (email, letter, or text message)

From a community health worker From a newspaper or radio

From social media Other

1a. Please specify other:

**Demographics / Características demográficas**

2. What is your race?

Mark one or more boxes AND print origins.

American Indian or Alaska Native Black or African American

Asian

Native Hawaiian or Other Pacific Islander White

Some other race Prefer not to answer

(Check all that apply)

2a.

Japanese Filipino Chinese Korean Other Asian

(Check all that apply)

2b.

Native Hawaiian Pacific Islander Samoan

Tongan Maori Fijian Chamorro Chuukese Kosraen

Marshallese Palauan Pohnpeian Yapese

Other Pacific Islander (Check all that apply)

2c. Specify other origin

3. Are you of Hispanic, Latino, or Spanish origin?	No, not of Hispanic, Latino, or Spanish origin

Yes, of Hispanic, Latino, or Spanish origin Prefer not to answer

3a. Please specify your origin	Mexican, Mexican Am., Chicano Puerto Rican

Cuban

Another Hispanic, Latino, or Spanish origin

3b. Please specify other Hispanic, Latino, or Spanish origin. For example, Salvadoran, Dominican, Colombian, Guatemalan, Spaniard, Ecuadorian, etc.

4. Age

For babies less than 1 year old, do not write the age	(Years) in months. Write 0 as the age.

5. What was your sex assigned at birth?	Female Male Intersex

None of these describe me Prefer not to answer

6. What terms best express how you describe your	Woman

gender identity?	Man

Non-binary

Transgender man/Female-to-male (FTM) Transgender woman/Male-to-female (MTF)

Gender non-binary/Genderqueer/Gender nonconforming Agender

Bigender

None of these describe me Prefer not to answer

7. Are you currently pregnant?	Pregnant

Not Pregnant Don't know

Prefer not to answer

8. Which of the following best represents how you	Gay

think of yourself at this time?	Lesbian

Straight; that is, not gay or lesbian, etc. Bisexual

None of these describe me Prefer not to answer

9. What is the highest level of education you have	Have never gone to school achieved outside or in the United States? Grades	5th grade or less

roughly equivalent to years of school.	6th to 8th grade

9th to 12th grade, no diploma

High school graduate or GED completed

Some college level/ Technical / Vocational degree Bachelor's degree

Other advanced degree (Master's, Doctoral degree) Prefer not to answer

Don't know

10. In 2019, what was your total household income	Less than $15,000 before taxes?	$15,000 - $19,999

$20,000 - $24,999

$25,000 - $34,999

$35,000 - $49,999

$50,000 - $74,999

$75,000 - $99,999

$100,000 and above Prefer not to answer

**Spoken Language / Lenguaje hablado**

11. Do you speak a language other than English at	Yes

home?	No

Prefer not to answer

12. What language(s)	Spanish

Vietnamese Mandarin Cantonese Tagalog Hawaiian Ilokano Navajo Other

12a. Specify other language(s)

**Housing / Vivienda**

Date of Housing, Employment and Insurance Collection

13. What best describes the people at your home:	Just me

Living with spouse, no kids Family including kids

Family with 3 generations (parents, children, grandchildren)

Family with 4 generations Living with roommates None of these

14. Are you currently living in transitional housing,	Yes

staying in a shelter, or experiencing homelessness?	No

Prefer not to answer Don't know

15. Do you live in any of these?	A group care setting Nursing home

Residential care facility for people with intellectual and developmental disabilities A psychiatric treatment facility

A group home

A board and care home Prison or jail

A halfway house Foster care

Homeless or in no consistent shelter Somewhere else

15a. Where do you stay/live?

**Now I am going to ask you a few questions about COVID-19 in your community. /**

**Ahora le voy a hacer algunas preguntas sobre la COVID-19 en su comunidad.**

16a. How many residents in NYCHA wear masks when they	All residents are outside of their apartments, but inside the	Most residents

building?	Less than half of residents

Few residents None

16b. Are youth and young adults (around the ages of	More likely 16-29) more or less likely to wear masks than other	Less likely adults in NYCHA?

17. How many residents in NYCHA maintain social	All residents

distancing (separate more than 6 feet) while in the	Most residents

building?	Less than half of residents

Few residents None

**Hardship as a result of COVID-19 / Dificultades debido a la COVID-19**

18. Have you, or has anyone in your household,	Yes

experienced a loss of employment income since the	No start of the COVID-19 pandemic (March 2020)?

19. We would like to know about what you do -- are you	Working now

working now, looking for work, retired, keeping house,	Only temporarily laid off, sick leave or maternity a student, or something else?	leave

Looking for work, unemployed Retired

Disabled, permanently or temporarily Keeping house

Student

Other (Specify) Prefer not to answer Don't know

19a(ii). As a student, are you also working?	Yes No

19a(i). Current employment status, Other - specify

19a. Are you considered an essential worker? An	Yes essential worker is someone who was required to go to	No

work even when stay at home orders were in place	Prefer not to answer

Unknown

19b. Would any of these describe where you work? If	Nursing care facilities

you work multiple jobs, select the closest match to	Visiting nurse or home health aide service your main job.	Building cleaning services

Public transportation Corrections facility

EMT or paramedic services Meat packing farm facility

Agriculture and food production facility Grocery store

Construction No

19c. If you were to test positive for COVID-19, would	Yes

you be able to isolate without losing your job?	No

Don't know

Prefer not to answer

19d. If you would be exposed to someone with COVID-19,	Yes would you be able to quarantine without losing your	No

job?	Don't know

Prefer not to answer

Date of Work PPE and Distancing Collection

19e. In your workplace, do you have access to	Yes, all of the time

necessary facilities to wash?	Yes, most of the time Some of the time Rarely

Not at all

19f. Does your work require you to be in close contact	Yes, all of the time (i.e. within 6 ft) with others?	Yes, most of the time

Some of the time Rarely

Not at all

19g. In your workplace, do you have access to	Yes, all of the time

necessary personal protective equipment (PPE)?	Yes, most of the time Some of the time Rarely

Not at all

20. What is the primary kind of health insurance or	I do NOT have health insurance

health care plan that you have now?	Private (purchased directly or through Employment) Public (Medicare, Medicaid, Tricare)

Don't know

Prefer not to answer

(Exclude plans that pay for only one type of Service (such as, nursing home care, accidents, family planning, or dental care) and plans that only provide extra cash when hospitalized.)

21. Did you lose health coverage because of the	Yes

COVID-19 pandemic?	No

Prefer not to answer Don't know

The COVID-19 pandemic may cause challenges for some people, whether they get COVID-19 or not. In the past 6 months have you or your family experienced any of the below challenges?

22. Getting the health care I need (including for	No, not a challenge

mental health)	Yes, a minor challenge

Yes, this is a major challenge

23. Having a place to stay/live	No, not a challenge Yes, a minor challenge

Yes, this is a major challenge

24. Getting enough food to eat	No, not a challenge Yes, a minor challenge

Yes, this is a major challenge

25. Having clean water to drink	No, not a challenge Yes, a minor challenge

Yes, this is a major challenge

26. Getting the medicine I need	No, not a challenge Yes, a minor challenge

Yes, this is a major challenge

27. Getting to where I need to go	No, not a challenge Yes, a minor challenge

Yes, this is a major challenge

28. Having adequate social support	No, not a challenge Yes, a minor challenge

Yes, this is a major challenge

29. Maintaining mental and emotional health	No, not a challenge Yes, a minor challenge

Yes, this is a major challenge

Food Insecurity:

I'm going to read you two statements that people have made about their food situation.

Please tell me whether the statement was OFTEN, SOMETIMES, or NEVER true for (you/you and the other members of your household) in the last 12 months.

30. "The food that (I/we) bought just didn't last, and	Often true

(I/we) didn't have money to get more."	Sometimes true Never true Don't know

Prefer not to answer

31. "(I/we) couldn't afford to eat balanced meals."	Often true Sometimes true Never true Don't know

Prefer not to answer

**Self-reported Health / Salud autonotificada**

32. Would you say your health in general is excellent,	Excellent very good, good, fair, or poor?	Very good

Good Fair Poor

Prefer not to answer Don't know

33. Do you have a disability that interferes with your	Yes

ability to carry out daily activities? Examples of	No

daily activities include walking, climbing stairs,	Prefer not to answer shopping, balancing a checkbook, bathing or dressing.

34. Are you currently homebound? Meaning you are	Yes

unable to leave your home or find it difficult to do	No so.

Date of Health Status Collection

35. How tall are you without shoes?	Feet and inches

Meters and centimeters

Please choose the units you would like to use for	Don't know

height.	Prefer not to answer

35a. Feet

35b. Inches

35a(i). Meters

| 35b(i). Centimeters |  | |
| --- | --- | --- |
|  |  |  |
| 36. Please choose the units you would like to use for weight | Kilograms Pounds |  |
| 37a. How much do you weigh without clothes or shoes? |  |  |
| If you are currently pregnant, how much did you weigh before your pregnancy? |  |  |
| 37a(i). How much do you weigh without clothes or shoes? |  |  |
| If you are currently pregnant, how much did you weigh before your pregnancy? |  |  |
| **Conditions / Condiciones** |  |  |
| Date of Medical History Collection |  |  |
|  |  |  |
| Do you have any of the following conditions? (Select all that apply) |  |  |
| 38. Immunocompromised condition | Yes | No |
| 39. Autoimmune disease | Yes | No |
| 40. Hypertension (HTN, high blood pressure) | Yes | No |
| 41. Diabetes | Yes | No |
| 42. Chronic kidney disease (CKD) | Yes | No |
| 43. Cancer diagnosis and/or treatment within the past 12 months | Yes | No |
| 44. Cardiovascular disease (CVD or heart disease) | Yes | No |
| 45. Asthma | Yes | No |
| 46. Chronic obstructive pulmonary disease (COPD) | Yes | No |
| 47. Other chronic lung disease | Yes | No |
| 48. Sickle Cell Anemia | Yes | No |
| 49. Depression | Yes | No |
| 50. Alcohol or substance use disorder | Yes | No |
| 51. Intravenous drug use | Yes | No |

52. Other mental health disorder	Yes	No
53. Other chronic condition	Yes	No

**Missed medical procedure / Procedimiento médico no realizado**

54. Since the start of the COVID-19 pandemic (March 2020), have you needed to postpone any medical care?

Yes	No

**Tobacco and Marijuana use / Consumo de tabaco y marihuana**

Date of Alcohol/Tobacco Use Collection

55. In your entire life, have you had at least 1 drink	Yes

of any kind of alcohol, not counting small tastes or	No

sips?	Prefer not to answer

56. How often do you have a drink containing alcohol?	Never

Monthly or less

2-4 times a month 2-3 times a week

4 or more times a week

Don't know or refuse to answer

57. Do you now smoke cigarettes?	Every Day Some Days Not at all

Prefer not to answer Don't know

57a. How many years have you smoked?

57b. If you smoke every day, on average, how many cigarettes per day do you smoke?

58. Do you now use electronic cigarettes every day,	Every Day

some days, rarely, or not at all?	Some Days Rarely

Not at all

Prefer not to answer Don't know

Date of Testing Collection

Testing History - I just want to reiterate for these questions that all of the information we collect is confidential, and that you may choose not to answer a question at any point.

59. Have you ever been tested for COVID-19?	Yes No

Don't know

Prefer not to answer

59a. How many times have you been tested?

59b. What month did you have your most recent COVID-19	January test?	February

March April May June July August

September October November December

59c. What year did you have your most recent COVID-19	2019

test?	2020

2021

2022

2023

59d. How were you tested for your most recent test?	Nasal Swab

Throat Swab Blood Sample Saliva

59e. What was the result of your most recent COVID-19	Negative test?	Positive

Never obtained results Indeterminate

Don't know

Prefer not to answer

59f. Where have you gone to be tested? (Check all that apply)

Health and hospitals (H+H) site City MD

Pop-up site (temporary set up or tent) Mobile van

At your regular doctor's office

At another doctor's office or health clinic At a hospital

Other

59f(i). Please specify other:

59g. Did you experience any of the following challenges when getting tested? (Check all that apply)

Difficulty finding test sites Long wait times

Fear of catching COVID while at testing site Pain/difficulty/discomfort from swabbing Uncomfortable taking public transit or car service to and from testing site

Long turnaround for results or never received results

Treated badly by testing staff Other

None

59g(i). Please specify other:

59h. Did you have any of the following positive experiences when getting tested? (Check all that apply)

Multiple places to test Short wait times/fast lines Helpful testing staff

Safe environment

No discomfort in testing Convenient location Other

None

59h(i). Please specify other:

59i. Have you ever paid for COVID-19 tests?	Yes No

59j. Have you ever tested positive for COVID-19?	Yes No

Don't know

Prefer not to answer

59k. What month did you first test positive for	January

COVID-19?	February

March April May June July August

September October November December

59l. What year did you first test positive for	2019

COVID-19?	2020

2021

2022

2023

60. Since September 1st 2020, has there been a time	Yes

when you wanted to get a COVID test but did not get	No

it?	Don't know

Prefer not to answer

60a. Why did you not get a test? (Check all that apply)

Couldn't afford it

Heard there weren't enough tests so didn't try Did not know where to go for test

Was too scared to get the test

Would have to miss work to get the test Other

60a(i). Please specify other:

61. If you needed to be tested in the future, where would you go?

Health and hospitals (H+H) site City MD

Pop-up site (temporary set up or tent) Mobile van

At your regular doctor's office

At another doctor's office or health clinic At a hospital

Other

**Knowledge and opinions on testing / Conocimientos y opiniones sobre las pruebas**

**62. Have you had any of these symptoms during the past week?**

Fever or chills Cough

Shortness of breath or difficulty breathing

Lack of energy or general tired feeling

Muscle or body aches Headache

New loss of taste or smell

Sore throat, congestion or runny nose

Feeling sick to your stomach or vomiting, diarrhea

Abdominal Pain Skin Rash Other

Yes	No	Don't know

63. I know where I can get COVID-19 testing in my	Strongly disagree

community.	Disagree

Neither disagree or agree Agree

Strongly agree

63a. Please specify other:

64. It is easy to get tested for COVID-19.	Strongly disagree Disagree

Neither disagree or agree Agree

Strongly agree

65. If I get a negative test result, it means [check all that apply]:

I don't have to worry about getting COVID-19 I don't have COVID-19 now

I can be around others without giving the virus to them

I can be around others without getting the virus from them

(Check all that apply)

66. If I get a positive result, it means: [check all that apply]

I will need to be admitted to the hospital I will need to isolate myself from others I will need to take off work

(Check all that apply)

67. How confident are you that a negative test result	Not at all confident means that you do not have COVID-19?	Somewhat confident

Confident Very confident

68. How confident are you that a positive test result	Not at all confident means that you do have COVID-19?	Somewhat confident

Confident Very confident

**Vaccination / Vacunación**

Date of Vaccine Acceptance Collection

69. Have you ever received a flu vaccination?	Yes No

Don't know

69a. Have you received a flu vaccine in the last 12	Yes

months?	No

Don't know

70. Have you received a COVID-19 vaccine?	Yes No

Prefer not to answer Don't know

70a(i). Have you completed the COVID-19 vaccination	Yes course? Most COVID-19 vaccines require two shots.	No

Prefer not to answer Don't know

70a. How likely are you to get an approved COVID-19	Very likely vaccine when it becomes available?	Fairly likely

Not too likely Not at all likely Definitely not Don't know

Prefer not to answer Not applicable

70b. Why would you get a COVID-19 vaccine?

I want to keep my family safe

I want to keep my community safe I want to keep myself safe

I have a chronic health problem, like asthma or diabetes

My doctor told me to get a COVID-19 vaccine I don't want to get really sick from COVID-19 I want to feel safe around other people

I believe life won't go back to normal until most people get a COVID-19 vaccine

Other

(Check all that apply)

70b(i). Please specify other:

70c. Why would you NOT get a COVID-19 vaccine?

I'm allergic to vaccines I don't like needles

I'm not concerned about getting really sick from COVID-19

I'm concerned about side effects from the vaccine I don't think vaccines work very well

I don't trust that the vaccine will be safe

I don't believe the COVID-19 pandemic is as bad as some people say it is

I don't want to pay for it

I don't know enough about how well a COVID-19 vaccine works

Other

(Check all that apply)

70c(i). Please specify other:

71. How likely are your friends and family to want an	Very likely approved vaccine when it becomes available to them?	Fairly likely

Not too likely Not at all likely Definitely not Don't know

Prefer not to answer Not applicable

**72. How much do you trust each of these sources to provide correct information about COVID 19? (Select one response for each row.)**

**¿Cuánto confía usted en cada una de estas fuentes para proporcionar información correcta sobre la COVID 19? (Seleccione una respuesta para cada fila)**

|  | Not at all | A little | Somewhat | A great deal | Don't know |
| --- | --- | --- | --- | --- | --- |
| Your doctor or health care provider |  |  |  |  |  |
| Your faith leader |  |  |  |  |  |
| Your close friends and members of your family |  |  |  |  |  |
| People you go to work or class with or other people you know |  |  |  |  |  |
| News on the radio, TV, online, or in newspapers |  |  |  |  |  |
| Your contacts on social media |  |  |  |  |  |
| The U.S. government |  |  |  |  |  |
| The U.S. Coronavirus Task Force |  |  |  |  |  |
