# Supplementary material for: Association between trusted sources of information and COVID-19 protective behaviors: results from a longitudinal survey of adults living in New York City during the pandemic
Source: Front Public Health. 2026 Sep 9;14:1838172. doi: 10.3389/fpubh.2026.1838172 (PMC13597789; doi:10.3389/fpubh.2026.1838172)
Supplement: Supplementary file 2 [file Supplementary_file_2.docx]

RADx-UP Phase II: Follow-Up Survey

*Frequency of survey: 1/week, for 5 weeks (total of 5 surveys)*

***In the following questions, we ask about places you have spent indoors in different settings. By indoors, we mean spending time in buildings for 15 minutes or more.***

1. In the past week, have you gone to a workplace, school or volunteer site that is outside your home?
   a. Yes
   b. No
2. **[If yes to Q1]** Is this site indoors?
   a. Yes
   b. No
3. **[If yes to Q2]** How many days in the past 7 days did you go in?
   a. 1 day
   b. 2 days
   c. 3 days
   d. 4 days
   e. 5 days
   f. 6 days
   g. 7 days
4. In the past week, did you wear a mask in these settings at any time?
   a. Yes
   b. No
5. **[If yes to Q4]** How often did you wear a mask there?
   a. 100% of the time
   b. Most of the time
   c. Roughly half of the time
   d. Less than half of the time
   e. Never
6. **[If yes to Q2]** On average, in the past week how many people did you interact with there without a mask?
   a. None
   b. 1-5 people
   c. 6-10 people
   d. 11-20 people
   e. More than 20 people

--------------------------------------------------------------------------------------------------------------------------------------------

7. In the past week, outside of school or work, did you attend any large social events such as a concert, sports game, dance event, etc.?
   a. Yes
   b. No
8. **[If yes to Q6]** Were the events indoors, outdoors or did you attend events in both types of settings?
   a. Indoors only
   b. Outdoors only
   c. Both indoors and outdoors
9. **[If answer to Q7 is indoors]** Approximately how many people were at the indoor events?
   a. Less than 10 people
   b. 10-20 people
   c. 21-50 people
   d. More than 50 people
10. [If answer to Q7 is indoors] In the past week, did you wear a mask in these settings at any time?
    a. Yes
    b. No
11. **[If yes to Q10]** How often did you wear a mask at these events?
    a. 100% of the time
    b. Most of the time
    c. Roughly half of the time
    d. Less than half of the time
    e. Never
12. **[If answer to Q7 is outdoors]** Approximately how many people were at the outdoor events?
    a. Less than 10 people
    b. 10-20 people
    c. 21-50 people
    d. More than 50 people
13. **[If answer to Q7 is outdoors]** In the past week, did you wear a mask in these settings at any time?
    a. Yes
    b. No
14. **[If yes to Q13]** How often did you wear a mask at these events?
    a. 100% of the time
    b. Most of the time
    c. Roughly half of the time
    d. Less than half of the time
    e. Never

--------------------------------------------------------------------------------------------------------------------------------------------

15. In the past week, outside of work or school, have you socially spent time with MORE than 5 people at a time
    a. Yes
    b. No
16. **[If yes to Q12]** Did you socialize indoors?
    a. Yes
    b. No
17. **[If yes to Q13]** How many days in the past 7 days did you gather with groups of more than 5 people?
    a. 1 day
    b. 2 days
    c. 3 days
    d. 4 days
    e. 5 days
    f. 6 days
    g. 7 days
18. **[If yes to Q13]** In the past week, did you wear a mask in these settings at any time?
    a. Yes
    b. No
19. **[If yes to Q18]** How often did you wear a mask?
    a. 100% of the time
    b. Most of the time
    c. Roughly half of the time
    d. Less than half of the time
    e. Never

--------------------------------------------------------------------------------------------------------------------------------------------

20. To the best you can recall, how many people did you spend time indoors with other than family member or at work/school setting without a mask?
    a. None
    b. 1-5 people
    c. 6-10 people
    d. 11-20 people
21. In the past week, did you go out to a restaurant, coffee shop, bar or club?
    a. Yes
    b. No
22. **[If yes to Q17]** How many days in the past 7 days did you go to one of these?
    a. 1 days
    b. 2 days
    c. 3 days
    d. 4 days
    e. 5 days
    f. 6 days
    g. 7 days
23. **[If yes to Q17]** In the past week, did you wear a mask in these settings at any time?
    a. Yes
    b. No
24. **[If yes to Q23]** How often did you wear a mask there?
    a. 100% of the time
    b. Most of the time
    c. Roughly half of the time
    d. Less than half of the time
    e. Never
25. **[If yes to Q17]** On average, in the past week how many people did you sit with there without a mask?
    a. None
    b. 1-5 people
    c. 6-10 people
    d. 11-20 people
    e. More than 20 people

--------------------------------------------------------------------------------------------------------------------------------------------

26. Did you ride in the subway or take a bus in the past week?
    a. Yes
    b. No
27. **[If yes to Q21**] How many days in the past 7 days did you take public transit?
    a. 1 day
    b. 2 days
    c. 3 days
    d. 4 days
    e. 5 days
    f. 6 days
    g. 7 days
28. **[If yes to Q21]** In the past week, did you wear a mask in these settings at any time?
    a. Yes
    b. No
29. **[If yes to Q28]** How often did you wear a mask there?
    a. 100% of the time
    b. Most of the time
    c. Roughly half of the time
    d. Less than half of the time
    e. Never
30. Did you go to a store such as a supermarket, shopping mall, or retail store in the past week?
    a. Yes
    b. No
31. **[If yes to Q30]** How many days in the past week did you go to one of these?
    a. 1 day
    b. 2 days
    c. 3 days
    d. 4 days
    e. 5 days
    f. 6 days
    g. 7 days
32. **[If yes to Q31]** In the past week, did you wear a mask in these settings at any time?
    a. Yes
    b. No
33. **[If yes to Q32]** How often did you wear a mask there?
    a. 100% of the time
    b. Most of the time
    c. Roughly half of the time
    d. Less than half of the time
    e. Never

-------------------------------------------------------------------------------------------------------------------------------------------

34. Are there any other settings you were at in the past week that you were exposed to other people without a mask?
    a. Yes
    b. No
35. In the past week, how many times did you stay at home for 24 hours straight?
    a. 0 times
    b. 1 time
    c. 2 times
    d. 3 times
    e. 4 times
    f. 5 times
    g. 6 times
    h. 7 times
36. In the past week, was your social contact with people outside your home:
    a. A lot less than normal
    b. Somewhat less than normal
    c. About the same as normal
    d. More than normal
    e. A lot more than normal
37. In the past week, have you used an at-home testing kit for COVID-19 that was provided by this study?
    a. Yes
    b. No
38. **[If yes to Q27]** What was the result of your at-home COVID-19 test?
    a. Positive
    b. Negative
    c. Failed
    d. Lost
    e. Other
39. In the past week, has anyone else in your household used any of the at-home test kits for COVID-19?
    a. Yes
    b. No
40. **[If yes to Q29]** What was the result of your household member’s at-home COVID-19 test?
    a. Positive
    b. Negative
    c. Pending result
    d. Don’t know/refused to answer
41. COVID Test Collection Setting
    a. Clinic
    b. Drive through
    c. Home
    d. Mobile Unit
    e. Lab
    f. Mail in
    g. Community Location (i.e., church, school, community center, etc.)
    h. Other, specify
42. COVID Test Manufacturer and Test Name
    a. Flowflex
43. COVID Test Performed Location
    a. Clinic
    b. Drive through
    c. Home
    d. Mobile Unit
    e. Lab
    f. Mail in
    g. Community Location (i.e., church, school, community center, etc.)
    h. Other, specify
44. COVID Specimen Collector
    a. Self-collect
    b. Health Care Provider collected
    c. Other, specify
45. COVID specimen Type
    a. Anterior nasal swab
    b. Mid-turbinate nasal swab
    c. Nasopharyngeal swab
    d. Oropharyngeal swab
    e. Nasal lavage
    f. Saliva
    g. Sputum
    h. Whole blood
    i. Other, specify
46. COVID Study Setting
    a. Community health center
    b. Nursing home of long-term care facility
    c. Prison or correctional facility
    d. Public Housing
    e. Rural
    f. Urban
    g. School
    h. In-home
    i. Other, specify
47. COVID Test Method Target
    a. Antibody
    b. Antigen
    c. Nucleic Acid/PCR
    d. Nucleic Acid/Isothermal
    e. Molecular/host reponse
    f. Biochemical marker (e.g., pH)
    g. Other, specify
48. On average, how many hours of sleep do you get in a 24-hour period? [ XX]
